# Experimental dataset on aluminium wedge slamming: Measurements of acceleration, pressure, strain, and video data

**DOI:** 10.1016/j.dib.2024.110818

**Published:** 2024-08-10

**Authors:** Saeed Hosseinzadeh, Kristjan Tabri, Tarmo Sahk, Ruttar Teär

**Affiliations:** a Maritime Engineering Research Group, School of Engineering, University of Southampton, Southampton, UK; b Department of Civil Engineering and Architecture, Tallinn University of Technology, Tallinn, Estonia; c Marine Technology Competence Center, Tallinn University of Technology, Kuressaare, Estonia

**Keywords:** Experimental study, Free-fall water entry, Flexible fluid structure interactions (FFSI), Impact-induced loads, Structural responses

## Abstract

This paper presents data from three experimental campaigns investigating slamming loads on a three-dimensional non-prismatic aluminium wedge, complementing the original research article “Slamming loads and responses on a non-prismatic stiffened aluminium wedge: Part I. Experimental study [1].” The experiments were designed to investigate the effects of slamming loads on structural responses through a series of free-fall drop tests. These tests included wedges with stiffened and unstiffened bottom plates to examine the influence of flexural rigidity on hydroelastic slamming. The experimental setup utilized three accelerometers for vertical acceleration measurement, sixteen pressure sensors for slamming pressure capture, and twenty strain gauges for recording structural responses. Detailed information on wedge geometry, material properties, and test plans is provided. Symmetric impact tests were conducted at drop heights from 25 cm to 200 cm with two different wedge masses. Asymmetric impact tests were carried out at three drop heights with heel angles ranging from 5 to 25°. The dataset includes time histories of sensor records, the geometry of the wedge section, and video footage from various runs. This comprehensive data offers insights into the effects of water impact velocity, deadrise angle, wedge mass, and bending stiffness on hydrodynamic pressures and structural responses on V-shaped sections. The experiments provide a valuable benchmark for future slamming impact research, aiding in the refinement of experiments, validation of numerical methods, and enhancement of mathematical models.

Specifications Table

**Table:** 

Subject	Ocean and Maritime Engineering
Specific subject area	Hydroelastic slamming studies on a non-prismatic wedge section under both symmetric and asymmetric conditions.
Type of data	Table (.mat), CAD (.igs), Videos (.mp4). Raw, Analysed, Filtered.
Data collection	All experiments were carried out in the Tallinn University of Technology towing tank, with a length of 60 m, a breadth of 5 m, a water depth of 3 m, and a maximum carriage speed of 6 m s^-1^. An aluminium wedge section with a length of 1.5 m, resembling the fore body structure of a medium-speed small craft, was built for the free fall drop tests. The prepared test setup enabled a wide range of both symmetric [1,2] and asymmetric [3] experiments. The model was equipped with accelerometers, pressure sensors, and strain gauges. All sensor data were collected using an HBM CX22B–W data recorder module connected to the HBM proprietary CatmanEASY AP software. The experiments were filmed with cameras from different directions.
Data source location	Institution: Tallinn University of Technology, Marine Technology Competence Centre City/Town/Region: Kuressaare Country: Estonia
Data accessibility	Repository name: TalTech Data Repository [4] Data identification number: https://doi.org/10.48726/j0mys-2dr75 Direct URL to data: https://data.taltech.ee/records/j0mys-2dr75
Related research article	Hosseinzadeh, S., Tabri, K., Hirdaris, S., & Sahk, T. (2023). Slamming loads and responses on a non-prismatic stiffened aluminium wedge: Part I. Experimental study. Ocean Engineering, 279, 114510*.*https://doi.org/10.1016/j.oceaneng.2023.114510

## 1 Value of the Data

• This dataset provides comprehensive measurements of slamming loads and structural responses on a non-prismatic aluminium wedge, offering insights into the complex dynamics of slamming problems. The data covers a wide range of impact velocities, deadrise angles, and structural configurations, making it valuable for studying the effects of these parameters on hydrodynamic pressures and structural deformations.
• Researchers can use this data to validate and improve computational fluid dynamics (CFD) models and fluid-structure interaction (FSI) simulations. The simultaneous recordings of pressures and strains are particularly useful for benchmarking coupled numerical methods, enabling more accurate predictions of slamming phenomena in marine structures.
• The dataset includes experiments with both stiffened and unstiffened bottom plates, allowing researchers to investigate the influence of flexural rigidity on hydroelastic slamming. This aspect of the data is crucial for developing more refined mathematical models that account for structural flexibility in impact scenarios.
• Naval architects and ship designers can use this data to optimize hull designs and improve the structural integrity of high-speed vessels, considering critical dynamic phenomena such as local slamming, whipping, and springing. The asymmetric impact tests, in particular, provide valuable information for assessing slamming loads in realistic sea conditions where perfect symmetry condition is rare.
• The presented dataset provides valuable insights into the practical limitations and challenges of slamming tests, serving as a guide for researchers planning future free-fall drop tests. The detailed documentation of experimental setup, sensor placement, and test conditions allows others to replicate or improve upon these methods. Researchers can learn from the strategies employed to mitigate issues such as sensor calibration, noise reduction, and ensuring repeatable impact conditions, thereby enhancing the quality and reliability of future slamming experiments across the field.

## 2 Background

Water slamming is a complex fluid-structure interaction phenomenon that occurs due to large relative motions between a structure and the water surface over a very short period of time in extreme weather conditions [[5], [6], [7]]. This highly nonlinear phenomenon, involving intricate air-water-structure interactions, poses a significant threat to the structural integrity of marine structures. The water entry impact problem has been extensively studied across various applications. Most previous research has focused on rigid body slamming. However, impact-induced loads can cause structural deformations, necessitating the investigation of simultaneous fluid-structure coupling [[8], [9], [10]]. Despite extensive studies on water slamming problems, the effect of elasticity on ship structures requires further investigation to gain deeper insights into the problem. Limited experiments have been conducted to examine the impact of hydrodynamic loads on structural deformations [[11], [12], [13], [14]]. High-quality experimental data on slamming loads and structural responses are essential for improving existing mathematical models and validating numerical simulations [[15], [16], [17], [18]]. Such data will enhance our understanding of fluid-structure interaction dynamics, contributing to the development of more resilient and safer marine structures. This study aims to provide detailed experimental data on the acceleration, pressure, and strain responses of a flexible aluminium flexible wedge, thereby offering valuable insights into the elastic effects during slamming impacts.

## 3 Data Description

The data from three experimental campaigns are presented in this article. The dataset includes MATLAB (.mat) files, the geometry of the model, and videos recorded during the tests. The data are organised into four folders: Symmetric, Asymmetric, CAD, and Videos. The test plans for each experimental campaign are detailed in Table 1, Table 2, Table 3, corresponding to symmetric impact with a lighter wedge, symmetric impact with a heavier wedge, and asymmetric impact with a lighter wedge, respectively. The free-fall drop test matrix was designed to align with the research objectives presented in related research works available in [[1], [2], [3]]. During the symmetric experiments, the average water temperature was measured at 17.0 °C and the air temperature at 19.4 °C. Fresh water properties were calculated according to ITTC - Recommended Procedures [19], resulting in a model freshwater density of 998.77 kg/m^–3^ and a model fresh water kinematic viscosity of 1.0811E-06 m²/s. The asymmetric experiments were conducted with water at 15.0 °C and air at 18.0 °C. Accordingly, the model freshwater density was 999.10 kg/m^–3^ and the model fresh water kinematic viscosity was 1.1386E-06 m²/s.

### 3.1 Symmetric slamming

The folder named “Symmetric” contains two subfolders: “m1” and “m2”, which hold data for symmetric impact tests. The “m1” folder includes 9 .mat files, representing data from experiments with various drop heights ranging from 25 cm to 200 cm. Details of each run are presented in Table 1. Each .mat file contains 6 tables with recorded data from different sensors. The table named “Acceleration” contains 4 columns, presenting the time histories of acceleration at different locations (aft, middle, and fore) of the wedge. This data was recorded using piezoelectric accelerometers. The columns are named as follows: time, A_a, A_m, and A_f, where acceleration values are given in units of ‘g’ (with 1 *g* = 9.81 m/s^2^). In addition to the high-frequency accelerometers, a six-degree-of-freedom inertial measurement unit (IMU) was employed to collect acceleration data at the mid-section of the wedge. The recorded data from this sensor is located in the table named “Motion”. The IMU acceleration data was used to calculate the vertical velocity and displacement of the wedge during free-fall impact, using cumulative trapezoidal numerical integration. The “Motion” table presents time histories of A (acceleration), V (velocity), and D (displacement).

**Table 1:** Test plan for experiments in symmetric conditions with light wedge (m1).

Run no.	Drop height	File name	Wedge weight	Test condition
1	25 cm	H025_m1.mat	55 kg	Symmetric
2	50 cm	H050_m1.mat		
3	75 cm	H075_m1.mat		
4	100 cm	H100_m1.mat		
5	125 cm	H125_m1.mat		
6	150 cm	H150_m1.mat		
7	175 cm	H175_m1.mat		
8	200 cm	H200_m1.mat		

In this series of experiments, sixteen pressure sensors were used to measure the impact pressure. To compare the pressure results of unstiffened and stiffened bottoms, the sensors were mounted symmetrically on the bottom of the wedge (eight sensors on the stiffened bottom and eight sensors on the unstiffened bottom). The pressure sensors (referred to as P) are labelled differently on the stiffened and unstiffened bottoms of the wedge. The recorded pressure data are located in tables named “Pressure_U” and “Pressure_S”, which represent the pressure data for the unstiffened and stiffened bottoms, respectively. The tables “Pressure_U” and “Pressure_S” include 9 columns presenting the time histories of pressure data on the unstiffened and stiffened bottoms of the wedge. For instance, P1_U presents the data from the first pressure sensor on the unstiffened bottom, and P1_S presents the data from the first pressure sensor on the stiffened bottom. All pressure data presented in this dataset are in kPa.

In addition to the pressure sensors, twenty linear strain gauges were installed on the inner side of the wedge to measure the structural response. The strain data are presented in the tables named “Strain_1” and “Strain_2”. These tables are separate because different data acquisition systems were used for some gauges, resulting in varying sampling rates. Similar to the pressure sensors, the strain gauges (denoted as S) were installed symmetrically on both the unstiffened and stiffened bottoms of the wedge. To measure strain distribution in different directions, twelve strain gauges were positioned transversely (denoted as T) and eight longitudinally (denoted as L). The U subscript indicates sensors on the unstiffened bottom, while the S subscript represents sensors on the stiffened bottom. For example, the column named S2T_U shows the data from the second transverse strain gauge on the unstiffened bottom, and S2T_S shows the data from the same gauge on the stiffened bottom. Similarly, S1L_U and S1L_S present the longitudinal strain data on the unstiffened and stiffened bottoms, respectively. All strain responses are presented in µm/m. Fig. 3 clearly shows the arrangement of all sensors, including accelerometers, pressure sensors, and strain gauges used during symmetric free fall impact.

The subfolder “m1” includes another .mat file named “PressurePeakData_m1”, which contains the maximum values of all pressure sensors for different drop heights in symmetric condition. This file includes two tables: “Pressure_unstiffened” and “Pressure_stiffened”. The columns name in these tables correspond to the sensors name previously explained. To maintain consistent data description and facilitate reuse, the measured data for the heavier wedge are organized similarly to the lighter wedge. The data for the heavier wedge are presented in the subfolder named “m2”, which contains data from 4 runs and a file of the maximum pressure values (“PressurePeakData_m2”). The details of the file names and drop heights are presented in Table 2.

**Table 2:** Test plan for experiments in symmetric conditions with heavier wedge (m2).

Run no.	Drop height	File name	Wedge weight	Test condition
1	25 cm	H025_m2.mat	82.5 kg	Symmetric
2	50 cm	H050_m2.mat		
3	75 cm	H075_m2.mat		
4	100 cm	H100_m2.mat		

### 3.2 Asymmetric slamming

The data for asymmetric impact tests are available in the folder named “Asymmetric”. This folder contains the measured parameters of twelve runs in total. The test plan for the asymmetric experiments, including initial drop heights, heel angles, and file names, is available in Table 3. It is worth noting that the arrangement of pressure sensors has been changed compared to the symmetric experiments. Fig. 4 shows the sensor arrangements for this series of tests. In the asymmetric condition, 12 pressure sensors were used on the unstiffened bottom, and 4 pressure sensors were used on the stiffened bottom. The sensors were arranged in this manner because the unstiffened bottom is the windward side and is the primary part in contact with the water surface. The test runs are named such that the first part of the name represents the drop height, and the second part indicates the angle of the wedge at a 30-degree deadrise angle with the water surface. For example, the file name H050_A25.mat includes the data of the test at a 50 cm drop height and a 5-degree heel angle. Each .mat file of asymmetric experiments includes four tables named “Pressure”, “Strain”, “Acceleration”, and “Acce_MTi”. The pressure and strain measurements are presented in kPa and µm/m, respectively. The columns of the tables are named the same as the sensors, making it easier to identify each one. The measurements from three piezoelectric accelerometers are stored in the “Acceleration” table. The columns “A_a”, “A_m”, and “A_f” are recorded directly from the sensors; however, the recorded acceleration values are divided into x and y components to assess the vertical and horizontal acceleration and are presented as A_ax and A_ay, for example. Additionally, the acceleration data measured by the IMU device is presented in the “Acce_MTi” table. All accelerations are presented in m/s^2^. Additionally, the maximum values of the pressure sensor measurements are presented in the file “PressurePeakData_Asym”.

**Table 3:** Test plan for experiments in asymmetric conditions.

Run no.	Drop height	Heel angle	File name	Wedge weight	Test condition
1	25 cm	5°	H025_A25.mat	55 kg	Asymmetric
2		10°	H025_A20.mat		
3		15°	H025_A15.mat		
4		20°	H025_A10.mat		
5		25°	H025_A5.mat		
6	50 cm	5°	H050_A25.mat		
7		10°	H050_A20.mat		
8		15°	H050_A15.mat		
9		20	H050_A10.mat		
10		25°	H050_A5.mat		
11	100 cm	5°	H100_A25.mat		
12		10°	H100_A20.mat		

To ensure consistency and reduce uncertainty in future numerical simulations, it is essential to use the same geometry employed in the tests. The geometry file is stored in a folder named “CAD.” This folder includes a file named “Wedge Geometry.IGS,” which can be utilised for future simulations. The availability of this file not only aids in the reproducibility of these experiments but also facilitates accurate comparisons between experimental and numerical data, enhancing the overall reliability and validity of the research outcomes. To illustrate the experimental procedure, video footage is provided in the folder named “Videos”. This folder contains four .MP4 files, each displaying footage from different runs. Each filename reflects the run number and test condition. For example, ‘H100.MP4’ features the video of run H100_m1 in a symmetric impact, while ‘H100_A20.MP4’ shows the video of run H100_A20 in an asymmetric impact. Additionally, the video named ‘H175_Underwater.MP4’ presents the underwater view of run H175_m1.

Ensuring the reliability of the data through repeatability tests provides confidence in the experimental results. The repeated trials help to identify any inconsistencies and confirm that the measurements are consistent across multiple tests. By analysing the standard deviation and relative standard deviation, the variability in the data can be quantified, providing insights into the precision of the measurements. This rigorous approach to data validation underscores the robustness of the experimental findings and enhances their credibility for further research and application.

**Fig. 1:**
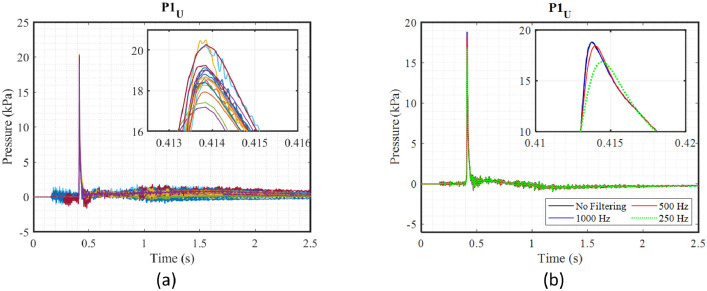
An example of data processing for the case under symmetric impact with a 25 cm drop height: a) repeatability of pressure measurements across 18 runs; b) the effect of different low-pass Butterworth filter rates on pressure data [1].

To enrich the measurements obtained during these three experimental campaigns, it is essential to present the repeatability and uncertainty of the data. To investigate the repeatability of the experimental measurements, eighteen trials were carried out for the first test case (H025_m1), and the rest of the cases were repeated at least twice. As already stated, the presented data are filtered and analysed. The details of the filtering process for different sensors can be found in [1]. Fig. 1 shows the results of repeated tests and the filtered pressure data as an example. Additionally, the mean values of the maximum accelerations, pressures, and strain measurements, as well as the standard deviation and relative standard deviation, are all presented in [1].

## 4 Experimental Design, Materials and Methods

All experiments were conducted at the Marine Technology Competence Centre (MARTE) of Tallinn University of Technology, using a towing tank measuring 60 m in length, 5 m in width, and 3 m in depth. The drop test setup was designed to accommodate a wide range of experiments, including both symmetric and asymmetric impacts. The test tower was constructed using Norcan 45×90 mm anodized aluminium profiles and Norcan 88×43 mm corner triangles. Two linear guide rails (HepcoMotion 44–1–1796) were attached to the front and back of the test frame to enable vertical free-fall motion. A manually operated winch was mounted on the ceiling, aligned with the centre axis of the test rig, to lift the wedge to the correct drop height during the experiments. Symmetric condition drop tests were conducted at various heights, ranging from 25 cm to 200 cm. For asymmetric impact tests, the test tower was modified to accommodate a wide range of heel angles. An angle adjustment mechanism was incorporated at the fore and aft of the wedge to set the desired heel angle. This test tower allowed for easy adjustment of drop height and heel angle (ranging from 5 to 25°) for conducting asymmetric wedge slamming experiments. The construction and installation of the test tower within a segment of the towing tank were executed with a focus on rigidity to minimize its impact on experimental results. Detailed descriptions of the symmetric test frame can be found in [1], and the asymmetric test tower in [3].

**Fig. 2:**
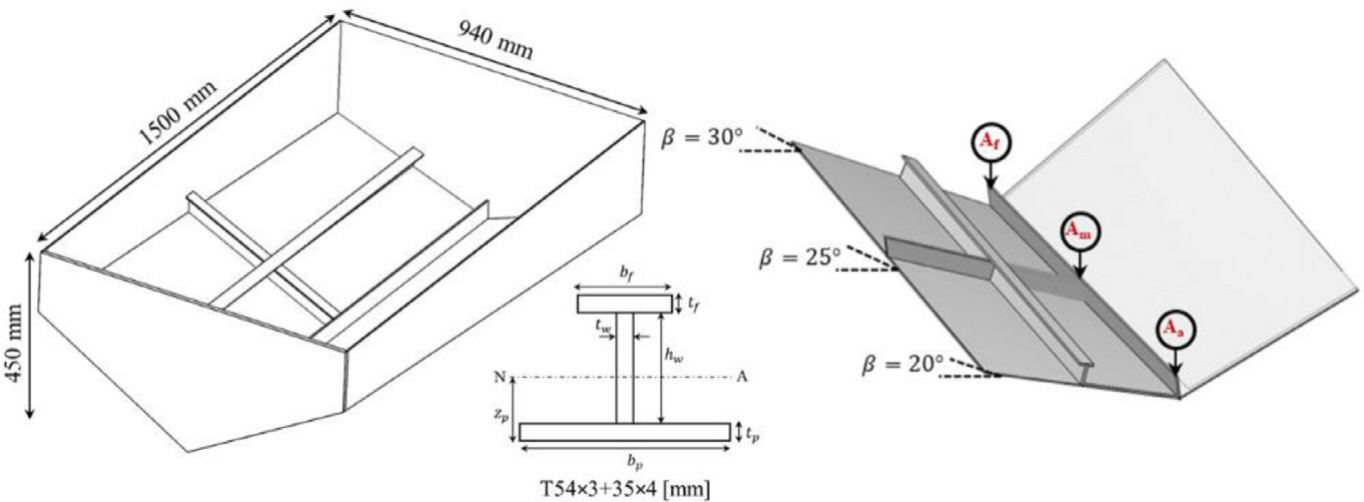
Drawing of the wedge with its dimensions, the stiffener configuration, and the location of the accelerometers at different deadrise angles [1,3].

A non-prismatic aluminium (alloy 5083-H111) wedge section was designed to examine the slamming pressure and structural responses during these experiments. The material properties of the wedge are provided in Table 4. The principal dimensions of the wedge are 1500×940×450 mm, featuring a V-shaped bottom with different structural flexibilities on each side. The wedge design aimed to balance practicality for laboratory-scale production and sufficient stiffness disparity between the port and starboard sides. To achieve this, the bottom of the specimen was intentionally designed with varying deadrise angles, ranging from 20 to 30° (Fig. 2). The starboard side's bottom, referred to as the “stiffened bottom”, consists of a 4 mm thick extruded aluminium panel, reinforced with a single T-shaped longitudinal stiffener and a transverse stiffener welded to the mid-bottom plate. The detailed dimensions of these stiffeners are listed in Table 4. In contrast, the port side of the wedge, known as the “unstiffened bottom,” lacks stiffeners and is also 4 mm thick. The fore and aft ends of the wedge are fitted with 10 mm thick end plates, and the keel is made of a 60×5 mm flat bar that is vertically welded to the apex of the wedge. An additional frame on top of the wedge provides extra stiffness to the sides and allows for a loop shackle attachment to hoist the wedge into the test tower. The wedge was tested with two different masses, 55 kg (m1) and 82.5 kg (m2), including all sensors, screws, welding, and the top frame (as detailed in Table 4).

**Table 4:** Material properties of the aluminium wedge section [1].

Density ($\rho_{Al}$)	2700 [kg m^-13^]
Modulus of Elasticity (E)	68 [GPa]
Shear Modulus (G)	26 [GPa]
Poisson Ratio (ϑ)	0.33
Tensile Strength	300 [MPa]
Longitudinal and Transverse Stiffener	T54×3 + 35×4 [mm]
Keel flat bar	60×5 [mm]
Thickness of Bottom plates	4 [mm]
Thickness of Endplates	10 [mm]
Thickness of Side plates	4 [mm]
Mass of the wedge (m1)	55 kg
Mass of the wedge (m2)	85.5 kg

**Fig. 3:**
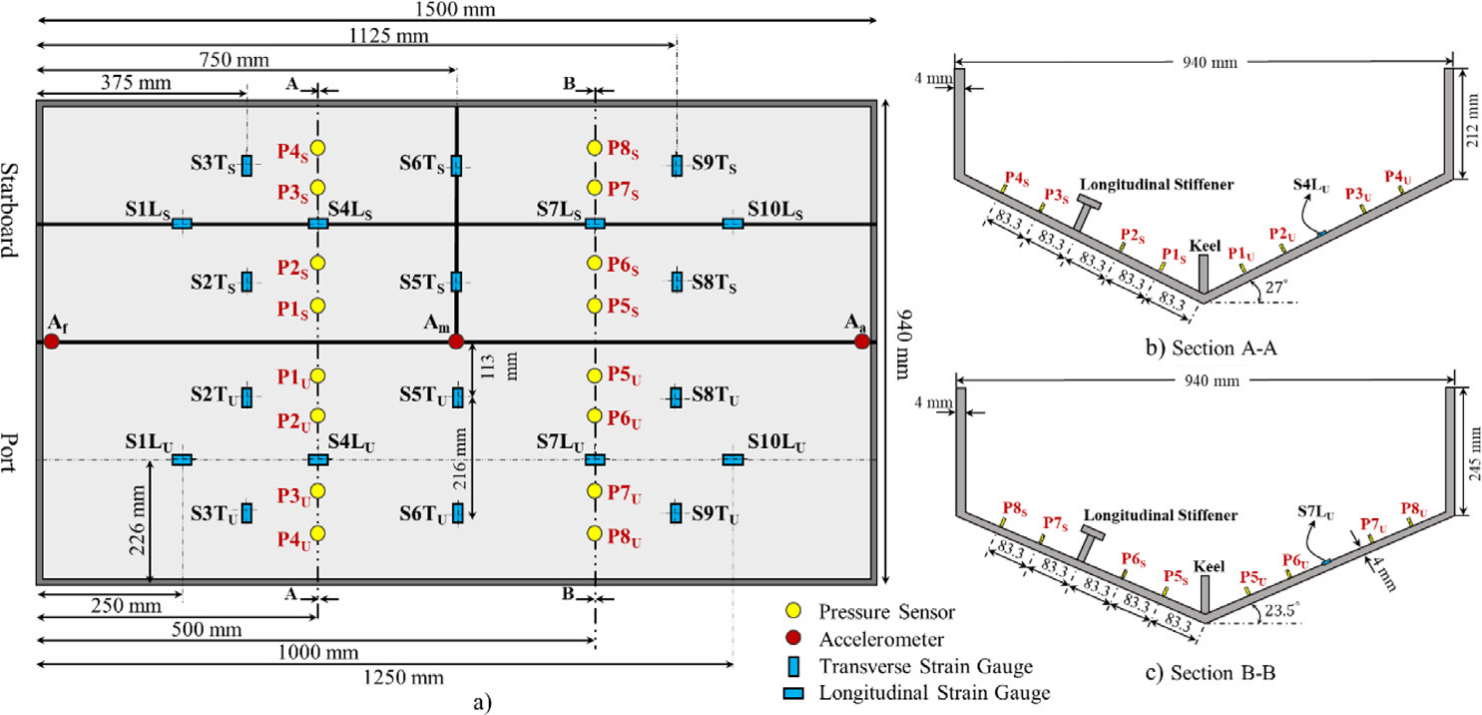
Locations and arrangements of sensors in symmetric impact: a) top view of the wedge showing the distribution of pressure sensors, strain gauges, and the accelerometers; b) A-A cross section indicating the location of pressure sensors at β=27°; c) B-B cross section indicating the location of pressure sensors at β=23.5° [1].

Fig. 3 details all the sensors used in these three experimental campaigns. Acceleration was measured at three different locations on the wedge section to analyse the influence of the deadrise angle. Three Dytran 3176B piezoelectric accelerometers were installed on the top of the keel. These accelerometers were positioned at the fore, middle, and aft of the section, denoted as A_f_ (β=30°), A_m_ (β=25°), and A_a_ (β=20°), respectively. The accelerometers were paired with a signal-conditioning module containing a TEDS (Transducer Electronic Data Sheet) chip, which stores the calibration data for the sensors. During the trial tests, it was found that the high frequency 50 g accelerometer could not capture the free-fall motion (pre-impact) due to the sensor's bandwidth [20] and showed some inconsistencies. Consequently, an Inertial Measurement Unit (XSens MTi-300 IMU) was also attached to the middle of the keel to measure the pre-impact motion. The measurement range of this sensor is 20 g.

To measure the slamming pressure, sixteen piezoelectric pressure transducers were arranged along the wedge surface. In this series of experiments, PCB-CA102B18 miniature dynamic pressure sensors with a resolution of 0.007 kPa and a measurement range of 344.7 kPa were used. The mounting thread length on the pressure sensor is 7.9 mm, and since the bottom plate is 4 mm thick, different spacers were employed. The pressure sensor has a measurement diameter of 8.6 mm. To ensure no leaks between the sensor threads and the mounting hole, Medium Strength Loctite thread glue was applied to all threads. As shown in Fig. 3, the pressure sensors are labelled differently on the port and starboard sides, as introduced in the previous section. To examine the three-dimensional effect, sensors were placed along two different characteristic lines, with a 500 mm longitudinal distance on each side. The first sensor, P1_U, is located 83.3 mm away from the keel, and the others are installed at increments of 83.3 mm. Pressure sensors on the port and starboard sides were mounted in symmetrical positions to compare their measured results with each other. Fig. 3, Fig. 3 show the locations of the pressure sensors on the wedge section with deadrise angles of 27° and 23.5°, respectively.

In addition to the accelerometers and pressure sensors, twenty linear strain gauges (HBM-1-LY13–6/120) were installed on the inner side of the bottom plates to measure the strain response of the wedge. To ensure a flat surface and enhance adhesion between the strain gauges and the wedge surface, the installation areas were wet sanded to 400 grits. Fig. 3a illustrates the arrangement of the strain gauges (denoted as S). To capture the strain distribution in different directions on the structure, twelve strain gauges were symmetrically positioned in the transverse direction and eight in the longitudinal direction. The strain gauges are labelled differently for the stiffened and unstiffened bottom of the wedge, and all strain gauges are detailed in the data description section.

**Fig. 4:**
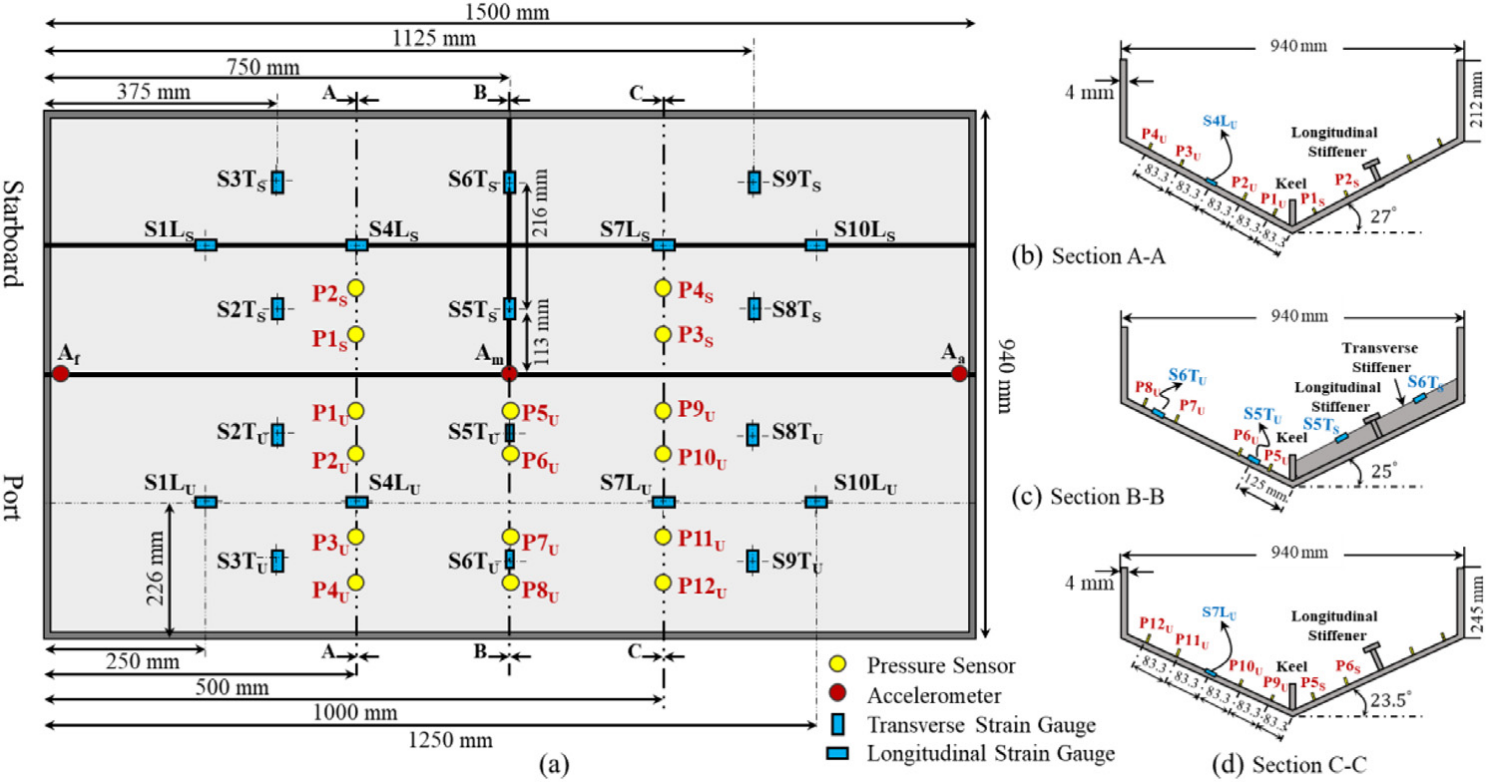
Locations and arrangements of sensors in asymmetric impact: a) top view of the wedge showing the distribution of pressure sensors, strain gauges, and accelerometers; b) A-A cross section indicating the location of pressure sensors at β=27°; c) B-B cross section indicating the location of pressure sensors at β=25°; d) C—C cross section indicating the location of pressure sensors at β=23.5°.

To obtain more useful data from the asymmetric condition experiments, the arrangement of the pressure sensors was changed. However, the locations of the accelerometers and strain gauges remained the same as in the symmetric impact tests. Fig. 4 presents the arrangement of all sensors used during the asymmetric tests. In the asymmetric impact tests, twelve pressure sensors were located on the unstiffened bottom, while four were used on the stiffened bottom. Three cross-sectional views of the wedge provide detailed locations of each sensor at different deadrise angles (Fig. 4b, c, and d).

All sensor data were simultaneously collected using a data acquisition (DAQ) system at the computer station located on the towing carriage. The port side pressure data were recorded with two MX410B highly dynamic universal amplifiers, each featuring 4 channels with a sampling rate of 100 kS/s per channel. The starboard pressure data were gathered using an 8-channel MX840B universal amplifier, with a sampling rate of 40 kS/s per channel. Additionally, a 4-channel MX440B universal amplifier was used to collect high-frequency acceleration data. The strain gauge data were gathered with a bridge amplifier that sampled at 20 kS/s. All sensor data were gathered using an HBM CX22B-W data recorder module, connected to HBM's proprietary CatmanEASY AP software, which facilitates easy and, in some cases, automatic sensor configuration. FireWire cables were used to connect the amplifiers to the data recorder module. Since the IMU could not be directly connected to the data recording system due to incompatibility with the HBM CatmanEASY AP software, it was connected via USB to a separate laptop running MT Manager software, specifically designed by XSens for their sensors. The specifications of the DAQ system and the modules used in the experiment are shown in Table 5.

**Table 5:** Specifications of data acquisition system used in the experiments [1].

Module	Number of channel	Sampling rate per channel	Signal bandwidth	Measuring ranges
One MX840B universal amplifier	8	40 kHz	7.2 kHz	± 10 mV/V
Two MX440B universal amplifier	4	40 kHz	7.2 kHz	± 10 mV/V
Two MX410B highly dynamic universal amplifier	4	100 kHz	40 kHz	± 20 mV/V
One MX1615B strain gauge bridge amplifier	16	20 kHz	3 kHz	± 20 mV/V
One HBM CX22B-W data recorder	–	4 MHz for 56 Ch.	–	–

**Fig. 5:**
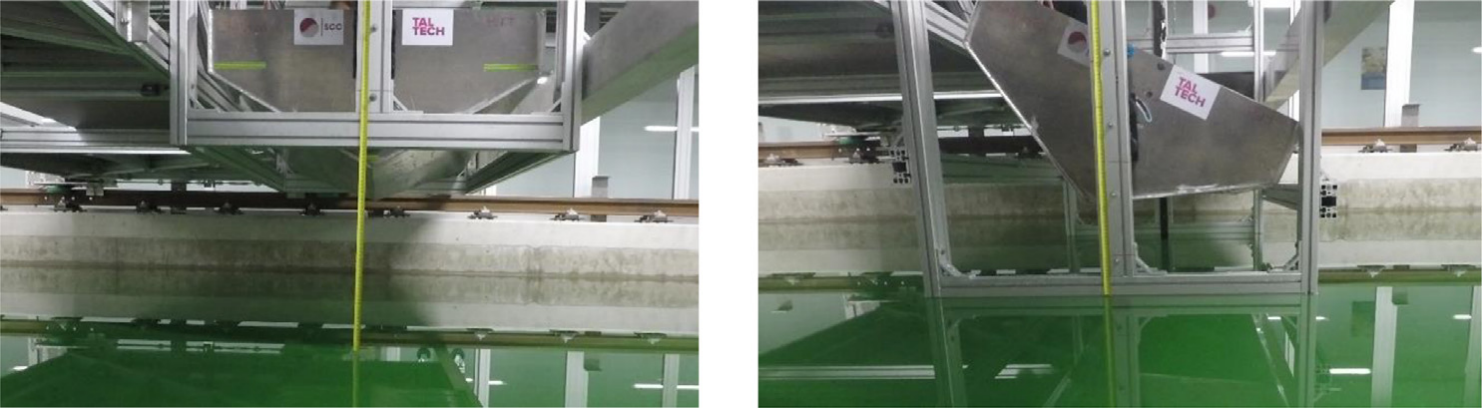
Initial position of the wedge: a) symmetric impact with a 50 cm drop height [1]; b) asymmetric impact with a 25 cm drop height and a 25-degree heel angle [3].

As stated, the symmetric free-fall drop tests were conducted at various heights ranging from 25 cm to 200 cm, in increments of 25 cm. To prepare for each test, the wedge was tied to the winch's shackle with nylon rope and hoisted to the desired height until it firmly touched the movable stopper. A measurement tape was attached to one side of the test rig to ensure proper positioning, and a laser level was used to accurately determine the drop height. Fig. 5a shows the initial position of the wedge at a height of 50 cm, as well as the test tower.

Before each test run, several important steps were followed. The correct drop height was verified using the laser level and measurement tape, and the wedge was carefully positioned at this height. All instrument cables and sensors were checked, and petroleum jelly was reapplied to the pressure sensors to maintain their performance. All channels of the data recorder were reset, and a wait time of 30–60 min was observed between runs to allow the water surface to calm down. After each test, the CatmanEASY AP analysis module was used to review the experiment results, ensuring that all sensors had operated correctly, and all relevant data had been recorded.

The asymmetric experiments were conducted at various heel angles, as detailed in Table 3. At the beginning of each test, the wedge was positioned at the desired heel angle and then raised to the designated drop height. Determining the heel angle during the experiment is crucial due to the variable deadrise of the wedge section along its length. Consequently, the angle of attack differs along each characteristic line where a set of pressure sensors is installed. Notably, at different heel angles, the right and left sides of the wedge exhibit varying deadrise angles. The initial position of the wedge at a 25-degree heel angle is depicted in Fig. 5b, providing a visual representation of the experimental setup. This setup ensures that the heel angle is accurately maintained, which is vital for the validity of the test results. Throughout the experiments, careful attention was given to the placement and adjustment of the wedge to ensure precise measurements and consistent data collection.

## Limitations

This dataset provides valuable insights into the slamming behavior of aluminium wedges; however, there are some limitations to acknowledge:

• The experimental scope was confined to V-shaped structures with deadrise angles between 20 and 30°. The investigation focused solely on the elastic response of aluminium materials, excluding analysis of plastic deformation which may occur in extreme slamming events.
• At high impact velocities, phenomena such as aeration and cavitation can significantly influence hydroelastic slamming and structural responses; however, these effects were not examined in the current tests.
• The experimental setup did not incorporate advanced visualization techniques like Particle Image Velocimetry (PIV) or Digital Image Correlation (DIC), which could have provided more comprehensive data on fluid dynamics and structural deformation patterns.

## Ethics Statement

The authors have read and follow the ethical requirements for publication in Data in Brief and confirming that the current work does not involve human subjects, animal experiments, or any data collected from social media platforms.

## CRediT authorship contribution statement

**Saeed Hosseinzadeh:** Conceptualization, Methodology, Software, Validation, Formal analysis, Investigation, Visualization, Data curation, Writing – original draft, Writing – review & editing. **Kristjan Tabri:** Conceptualization, Methodology, Writing – review & editing, Supervision, Project administration, Funding acquisition. **Tarmo Sahk:** Data curation, Visualization. **Ruttar Teär:** Data curation, Visualization.

## Data Availability

Dataset in support of the paper ‘Experimental dataset on aluminium wedge slamming: measurements of acceleration, pressure, strain, and video data’ (Original data) (TalTech Data Repository).

## References

[1] Hosseinzadeh S., Tabri K., Hirdaris S., Sahk T. (2023). Slamming loads and responses on a non-prismatic stiffened aluminium wedge: Part I. Experimental study. Ocean Eng..

[2] Hosseinzadeh S., Tabri K. (2021). Developments in the Analysis and Design of Marine Structures.

[3] Hosseinzadeh S., Tabri K. (2024). Experimental study on the dynamic response of a 3-D wedge under asymmetric impact. J. Hydrodyn..

[4] Hosseinzadeh S., Tabri K., Sahk T., Teär R. (2024). Dataset in support of the paper ‘Experimental dataset on aluminium wedge slamming: measurements of acceleration, pressure, strain, and video data’ (Version v1) [Data set]. TalTech Data Reposit..

[5] Abrate S. (2011). Hull slamming. Appl. Mech. Rev..

[6] Faltinsen O.M. (2005).

[7] Hosseinzadeh S. (2023).

[8] Bereznitski A. (2001). Slamming: the role of hydroelasticity. Int. Shipbuild. Progress.

[9] Faltinsen O.M. (2000). Hydroelastic slamming. J. Mar. Sci. Technol..

[10] Hirdaris S., Temarel P. (2009). Hydroelasticity of ships: recent advances and future trends. Proc. Inst. Mech. Eng. Part M.

[11] Piro D.J., Maki K.J. (2013). Hydroelastic analysis of bodies that enter and exit water. J. Fluids Struct..

[12] Panciroli R., Porfiri M. (2015). Analysis of hydroelastic slamming through particle image velocimetry. J. Sound Vib..

[13] Tödter S., El Moctar O., Neugebauer J., Schellin T.E. (2020). Experimentally measured hydroelastic effects on impact-induced loads during flat water entry and related uncertainties. J. Offshore Mech. Arct. Eng..

[14] Spinosa E., Iafrati A. (2021). Experimental investigation of the fluid-structure interaction during the water impact of thin aluminium plates at high horizontal speed. Int. J. Impact Eng..

[15] Hosseinzadeh S., Izadi M., Tabri K. (2020). International Conference on Offshore Mechanics and Arctic Engineering.

[16] Hosseinzadeh S., Tabri K. (2021). Hydroelastic effects of slamming impact loads during free-fall water entry. Ships Offsh. Struct..

[17] Yan D., Hosseinzadeh S., Lakshmynarayanana A., Mikkola T., Hirdaris S. (2021). Of the 23rd Numerical Towing Tank Symposium.

[18] Hosseinzadeh S., Tabri K., Topa A., Hirdaris S. (2023). Slamming loads and responses on a non-prismatic stiffened aluminium wedge: Part II. Numerical simulations. Ocean Eng..

[19] ITTC (2011).

[20] Lewis S.G., Hudson D.A., Turnock S.R., Taunton D.J. (2010). Impact of a free-falling wedge with water: synchronized visualization, pressure and acceleration measurements. Fluid Dyn. Res..

